# Analysis on a database of ship accidents in port areas

**DOI:** 10.1016/j.dib.2023.109127

**Published:** 2023-04-11

**Authors:** Massimiliano Marino, Luca Cavallaro, Elisa Castro, Rosaria Ester Musumeci, Matteo Martignoni, Federico Roman, Enrico Foti

**Affiliations:** a Department of Civil Engineering and Architecture, University of Catania, Via Santa Sofia 64, Catania, 95123, Italy; b DNV Maritime Italy, Calata Molo Vecchio, 15, Genoa, 16128, Italy; c Department of Engineering and Architecture, University of Trieste, Via Alfonso Valerio, 6/1, Trieste, 34127, Italy

**Keywords:** Maritime safety, Risk assessment, Safe navigation, Collision avoidance

## Abstract

In the last 15 years, the number of vessels in the world fleet has grown by around 53% and their gross tonnage has increased by 47%, with a consequent significant increment of marine accidents worldwide.

Accident database are the basic resource for risk assessment methods to help decision-makers to enact strategies and undergo hazard and vulnerability mitigation measures. Understanding ship accidents distribution in terms of involved GT, typical age of the affected vessels, category of the ships, as well as distribution of underlying causes and consequences is the first necessary step to improve accident mitigation actions to be implemented for future assessments.

In the present work, the results of an analysis on a database of vessel accidents in Mediterranean and worldwide port areas developed within the framework of the project ISY PORT (Integrated SYstem for navigation risk mitigation in PORTs) is herein presented. The distribution of accidents was analyzed in terms of relevant vessel characteristics i.e. Grosse Tonnage (GT), age at the time of the accident, ship's category, causality event, weather conditions and number of fatalities/injuries/lost at sea. The database can be used as a basis for maritime risk assessment methods and for calibration of real-time ship collision avoidance scenarios.

**Specifications Table**

**Table:** 

Subject:	Ocean and maritime engineering
Specific subject area:	Maritime hazard and navigation risk assessment
Type of data:	Table
How the data were acquired:	Interrogation and filtering of SeaSearcher and IHS databases
Data format:	Raw Analyzed Filtered
Description of data collection:	The data were collected from the SeaSearcher and IHS databases.These two databases were compared considering IMO Number, accident date and location of the accident (at sea or in port), in order to create a unified database with consistent data and as much reliable as possible. In the event of common accidents found within the system (meaning with the same IMO number and the same accident date), the location was compared. If the two locations in the databases were not matching, the incident was not included in the unified database for the analysis. The database used to conduct the analysis (after the comparison) comprises of 13,846 incidents worldwide, of which 2,799 occurred in port area.
Data source location:	Mediterranean port areas within SeaSearcher and IHS database of accidents
Data accessibility:	Repository: Mendeley Data DOI: 10.17632/rwwfg3r5yc.3 Link: https://data.mendeley.com/datasets/rwwfg3r5yc
Related research article:	Marino, M., Cavallaro, L., Castro, E., Musumeci, R. E., Martignoni, M., Roman, F., & Foti, E. (2023). New frontiers in the risk assessment of ship collision. Ocean Engineering, 274, 113999. doi:10.1016/j.oceaneng.2023.113999

## Value of the Data

• The dataset provide insights on ship collision accidents in the Mediterranean and worldwide, obtained from the analysis of the IHS and SeaSearcher merged datasets.
• The provided data are intended to support calibration of ship collision risk assessment methodologies (e.g. Bayesian networks, neural networks, swarm intelligence), with a specific focus on waterways and port areas.
• The dataset addresses different type of accidents (heavy weather, fire…) to support risk assessment due to different type of threats (environmental, economic, climatic).

## 1 Objective

Understanding ship accidents causes and consequences is the first necessary step to improve accident mitigation strategies [1]. This can help to identify patterns and trends in accidents, which can be used to inform prevention measures and response plans. The aim of this dataset is to provide a statistical analysis of vessels accidents that have occurred in port areas. The dataset investigates the causes and consequences of these accidents, with a focus on worldwide and Mediterranean trends. By analyzing these data, it is possible to gain insights into the factors that contribute to accidents and evaluate their impacts.

## 2 Data Description

The dataset [2] includes the results of an analysis on a database of ship collision accidents in port areas occurred worldwide, from 1990 to Q1 of 2021. The analysis was also conducted only on Mediterranean port areas, in order to provide a comparison with worldwide statistics. The marine accidents were classified based on the following accident characteristics:

• Gross Tonnage (GT): the accidents were divided into 6 GT classes, namely:
  ■ 0 – 500 GT;
  ■ 501 – 5.000 GT;
  ■ 5.001 – 10.000 GT;
  ■ 10.001 – 20.000 GT;
  ■ 20.001 – 50.000 GT;
  ■ 50.001 -100.000 GT;
  ■ Above 100.001 GT.
• Age: the accidents were divided into 7 age classes, namely:
  ■ 0 – 5 years;
  ■ 6 - 10 years;
  ■ 11- 15 years;
  ■ 16 – 20 years;
  ■ 21 – 25 years;
  ■ 26 – 30 years;
  ■ Above 31 years.
• Ship Category: The accidents were divided according to the main ship categories (General cargo, bulk carrier, containership, tanker, Roll-on/roll-off passenger, Roll-on/roll-off, cruise ship and others);
• Causes:
  ○ Causality type: The accidents were divided according to the causality type (collision, contact, fire explosion, machinery damage/failure, Wrecked/stranded, miscellaneous, hull damage and foundered) as reported in the accident database;
• Marine weather conditions: Marine conditions at the time of the accident have been reported. The accidents were divided into 5 weather condition classes, namely:
  ■ Heavy weather;
  ■ Good visibility and good weather;
  ■ Fog/Mist/Poor visibility;
  ■ Freezing Conditions;
  ■ Hurricane.
• Consequences:
  ○ Accidents that have consequence for human life, and especially cases that reported:
    ■ Fatalities;
    ■ Injuries;
    ■ Lost at sea.
  ○ Loss of ship and/or cargo.

The results obtained with the segmentations reported above are reported in Table 1, Table 2, Table 3, Table 4, Table 5, Table 6.

**Table 1:** Distribution of accidents in port areas by gross tonnage.

GT	Mediterranean	Worldwide
0 - 500	0,0%	0%
500 – 5.000	39,6%	28,7%
5.000 – 10.000	13,6%	15,4%
10.000 – 20.000	14,2%	16,1%
20.000 – 50.000	23,0%	26,9%
50.000 – 100.000	7,3%	10,3%
100.000 +	2,4%	2,6%
Average	19.891	23.670
Median	8.337	13.239
Standard deviation	27.885	28.810

**Table 2:** Distribution of accidents in port areas by age of the ship at the time of the accidents.

Vessel's age	Mediterranean	Worldwide
0 - 5	16,9%	23,4%
5 - 10	16,6%	20,0%
10 - 15	13,6%	16,3%
15 - 20	15,5%	14,3%
20 - 25	15,5%	12,7%
25 - 30	13,9%	8,7%
30 +	7,9%	4,5%
Average	16	14
Median	16	13
Standard deviation	9,9	9

**Table 3:** Distribution of accidents in port areas by ship's category.

Ship's category	Mediterranean	Worldwide
General Cargo	26,7%	23%
Passenger ro/ro	24,4%	13%
Fully cellular containership	12,0%	15%
Tanker	10,4%	14%
Bulk Carrier	10,1%	18%
Roll On Roll Off	6,5%	8%
Cruise Ship	5,0%	3%
Other	4,9%	5%

**Table 4:** Distribution of accidents in port areas by causality event.

Causality event	Mediterranean	Worldwide
Collision	37,2%	31,2%
Contact	25,2%	29,3%
Fire/explosion	12,8%	12,2%
Machinery damage/failure	8,8%	9,4%
Wrecked/stranded	8,7%	9,3%
Miscellaneous	4,6%	5,3%
Hull damage	1,6%	3,3%
Foundered	1,1%	0,8%

## 3 Experimental Design, Materials and Methods

The database used in the analysis was created starting from the following two databases:

• SeaSearcher [3], which recorded 79,592 vessel accidents between 1967 and Q2 of 2021;
• IHS [4], which recorded 23,897 vessel accidents between 1990 and Q2 of 2021

**Table 5:** Distribution of accidents in port areas by weather conditions.

Weather conditions	Mediterranean	Worldwide
Unknown/Not Reported weather conditions	74,9%	76,9%
Heavy Weather	19,1%	14,5%
Good visibility and Good Weather	5,7%	6,3%
Fog/Mist/Poor Visibility	0,3%	1,5%
Hurricane Etc.	0%	0,6%
Freezing Conditions	0%	0,2%

**Table 6:** Number of fatalities, injured and lost at sea caused by accidents in the port areas.

		Mediterranean	Worldwide
Fatalities	Accidents without fatalities	2753	619
	Accidents with at least 1 fatality	46	15
	Accidents with more than 2 [1] fatalities	24	6
	Maximum number of fatalities for a single accident	10	4
	Total number of fatalities	108	24
Injured	Accidents without injured	2745	614
	Accidents with at least 1 injured	54	20
	Accidents with at more than 3 [1] injured	28	12
	Maximum number of injured for 1 accident	55	55
	Total number of injured	337	131
Lost at sea	Accidents without lost at sea	2795	633
	Accidents with at least 1 lost at sea	4	1
	Accidents with at more than 3 [1] lost at sea	2	1
	Maximum number of lost at sea for 1 accident	15	3
	Total number of lost at sea	22	3
Accidents with human life consequences [2]		31	95

[1] The value is defined as the median value

[2] The value is the number of the accident with human life consequences. In some cases, the accident has caused fatalities, injured and lost at sea at the same time. This value is not the sum of accident with at least 1 fatalities/injured/lost at sea.

These two databases were compared considering IMO Number, accident date and location of the accident (at sea or in port), in order to create a unified database with consistent data and as much reliable as possible. In the event of common accidents found within the system (meaning with the same IMO number and the same accident date), the location was compared. If the two locations in the databases were not matching, the incident was not included in the unified database for the analysis. The database used to conduct the analysis (after the comparison) comprises of 13,846 incidents worldwide, of which 2,799 occurred in port area.

The focus of the overview and analysis of the marine incidents is the Mediterranean area, but the analyses have also been conducted on the worldwide incidents database, for comparison and are presented in the article.

The incidents occurred in the Mediterranean ports, considering ship with an IMO number and with a gross tonnage (GT) above 500 GT from 1990 to Q2 of 2021 are 634, and the analyses presented in this article are based on this dataset. The incidents occurred in the ports worldwide, when considering the same constrains of vessels GT and IMO number as in the Mediterranean analysis, are 2,799 from 1990 to Q2 of 2021.

Each accident includes the following information: IMO, Accident date, Name of the vessels, Flag, Deadweight (DWT), Grosse Tonnage (GT), Age of the vessel, Class, Vessel type, Cause of accident, Loss of vessel/cargo, Pollution, Number of fatalities, Number of injured, number of lost at sea, Port of accident, location of accident, weather at time of accident.

## Ethics Statements

The present work meets the publisher ethical requirements (https://www.elsevier.com/authors/journal-authors/policies-and-ethics), and does not involve studies with animals and humans.

## CRediT authorship contribution statement

**Massimiliano Marino:** Conceptualization, Writing – original draft. **Luca Cavallaro:** Conceptualization, Writing – review & editing, Supervision. **Elisa Castro:** Formal analysis. **Rosaria Ester Musumeci:** Writing – review & editing. **Matteo Martignoni:** Data curation, Formal analysis. **Federico Roman:** Writing – review & editing. **Enrico Foti:** Supervision, Funding acquisition.

## Declaration of Competing Interest

The authors declare that they have no known competing financial interests or personal relationships that could have appeared to influence the work reported in this paper.

## Data Availability

Analysis on a database of ship accidents in port areas (Original data) (Mendeley Data).
